# B-type natriuretic peptide is a long-term predictor of all-cause mortality, whereas high-sensitive C-reactive protein predicts recurrent short-term troponin T positive cardiac events in chest pain patients: a prognostic study

**DOI:** 10.1186/1471-2261-8-34

**Published:** 2008-11-25

**Authors:** Trygve Brügger-Andersen, Volker Pönitz, Harry Staines, David Pritchard, Heidi Grundt, Dennis WT Nilsen

**Affiliations:** 1 Institute of Medicine, University of Bergen, Bergen, Norway; 2 Department of Medicine, Stavanger University Hospital, Stavanger, Norway; 3 Sigma Statistical Services, Balmullo, UK; 4 Axis-Shield, Dundee, UK

## Abstract

**Background:**

Few studies have addressed whether the combined use of B-type natriuretic peptide (BNP) and high-sensitive C-reactive protein (hsCRP) improves risk stratification for mortality and cardiovascular events in a population with chest pain and suspected acute coronary syndromes (ACS). Therefore, we wanted to assess the incremental prognostic value of these biomarkers with respect to long-term all-cause mortality and recurrent troponin T (TnT) positive cardiac events in 871 patients admitted to the emergency department.

**Methods:**

Blood samples were obtained immediately following admission.

**Results:**

After a follow-up period of 24 months, 129 patients had died. The BNP levels were significantly higher among patients dying than in long-term survivors (401 (145–736) versus 75 (29–235) pq/mL [median, 25 and 75% percentiles], p = 0.000). In a multivariable Cox regression model for death within 2 years, the hazard ratio (HR) for BNP in the highest quartile (Q4) was 5.13 (95% confidence interval (CI), 1.97–13.38) compared to the lowest quartile (Q1) and was associated with all-cause mortality above and beyond age, congestive heart failure and the index diagnosis ST-segment elevation myocardial infarction. HsCRP rendered no prognostic information for all-cause mortality. However, within 30 days, the adjusted HR for patients with recurrent TnT cardiac positive events hsCRP in Q4 was 14.79 (95% CI, 1.89–115.63) compared with Q1 and was associated with recurrent ischemic events above and beyond age, hypercholesterolemia and TnT values at admission.

**Conclusion:**

BNP may act as a clinically useful biomarker when obtained at admission in an unselected patient population following hospitalization with chest pain and potential ACS, and may provide complementary prognostic information to established risk determinants at long-term follow-up. Our data do not support the hypothesis that the additional assessment of hsCRP will lead to better risk stratification for survival than BNP alone.

**Trial registration:**

NCT00521976

## Background

B-type natriuretic peptide (BNP) is a counter-regulatory peptide hormone predominantly synthesized in the ventricular myocardium. BNP is released into the circulation in response to ventricular dilatation and pressure overload, and reflects ventricular wall stress and tissue hypoxia rather than cell injury per se [1,2]. It is a well known marker of left ventricular dysfunction and heart failure (HF), and it provides prognostic information beyond and above left ventricular ejection fraction (LVEF) in patients with acute coronary syndromes (ACS) [3]. This marker of neurohormonal activation plays a pivotal role across the spectrum of ACS, including patients with ST-elevation myocardial infarction (STEMI) and non ST-elevation myocardial infarction (NSTEMI). Moreover, elevated natriuretic peptides at presentation have been shown to identify patients with ACS who are at higher risk of death and HF and add information to that provided by the troponins [4-6].

C-reactive protein (CRP) is an acute-phase reactant that is produced in response to acute injury, infection or other inflammation stimuli. It is a marker for underlying systemic inflammation, and it has been suggested to play a role in the initiation and propagation of atherosclerosis and ultimately to plaque rupture and the ensuing thrombotic complication [7]. Elevated levels of CRP were first reported in patients hospitalized with ACS in the early 1990s and were shown to predict mortality [8,9]. Through the use of appropriate high-sensitive assays, it has been possible to investigate the relationship between plasma CRP levels that previously were considered to be normal and cardiovascular disease (CVD).

Nevertheless, it is still under debate which markers should be preferred for risk prediction [10,11]. It has been suggested that the combined evaluation of natriuretic peptides and CRP may yield incremental prognostic information in the risk stratification of patients with ACS [12,13], and their combined use has been shown to improve long-term risk prediction of mortality in patients with stable coronary heart disease (CHD) [14]. To our knowledge, there are limited data available that directly compare these two markers in a prospective manner in an unselected patient population presenting to the emergency department (ED) with chest pain. In addition, their role in risk stratification in patients with ACS is still under evaluation, and therefore additional investigations are necessary.

The aims of this study were to explore the ability of BNP and hsCRP to predict risk of death and recurrent troponin T (TnT) positive cardiac events within 24 months in a patient population hospitalized with chest pain and suspected ACS, and to evaluate the incremental prognostic value of these biomarkers for risk stratification in ACS.

## Methods

### Study Design and Patient Population

The study was a single-center prospective follow-up study (RACS: Risk in the Acute Coronary Syndrome) in which 871 patients with chest pain and potential ACS were consecutively admitted to the ED of Stavanger University Hospital, Stavanger, Norway from November 2002 to September 2003. The main exclusion criteria were age < 18 years or unwillingness or incapacity to provide informed consent and prior inclusion in the present study. Patients with missing data for BNP or hsCRP were excluded from the present analyses.

The primary outcome measure of the present study was all-cause mortality from the time of inclusion until 2 years follow-up. The secondary outcome included the combined endpoint of cardiac death or recurrent TnT positive cardiac events (TnT > 0.05 ng/mL and a pattern of gradual rise and fall in TnT) and a third endpoint consisted of recurrent TnT positive cardiac events.

Survival status, date and cause of death and clinical data were obtained by telephone interview at 30 days, 6, 12 and 24 months during the 2 year follow-up period. In case of incapacity to provide information, the general practitioner or nursery home were contacted to obtain relevant data. In addition, hospital journals were searched for confirmation of reported data.

Clinical and laboratory parameters, including assessment of previous MI, angina pectoris, CHF, diabetes mellitus (defined as either whole blood fasting glucose concentrations above 6.1 mmol/L, two hour post glucose load concentrations above 10.0 mmol/L or medical treated diabetes mellitus), smoking status (stratified in categories of current smokers, previous smokers or patients who never smoked), hypercholesterolemia (defined as total cholesterol concentrations above 6.5 mmol/L or medical treated hypercholesterolemia) and arterial hypertension (defined as repeated blood pressure measurements above 140/90 mmHg or treated hypertension) were based on hospital records and personal interviews.

Electrocardiographic (ECG) findings at admission were classified according to the presence of ST-segment changes (i.e. ST-segment depression or elevation, T-wave inversion or left bundle-branch block). The term ACS in the present study encompasses unstable angina (UAP), NSTEMI and STEMI. The following classification for the index diagnosis was used: STEMI; ST-segment elevation combined with TnT values > 0.05 ng/mL. NSTEMI; Transient ST-segment elevation, ST-segment depression, or T-wave inversion in at least 2 contiguous leads combined with TnT values > 0.05 ng/mL. UAP; Transient ST-segment depression or T-wave inversion and TnT values < 0.05 ng/mL. No-ACS; All other conditions (i.e. unspecific chest pain, arrhythmias, atrial fibrillation etc.) without ECG changes and with negative troponins.

Written informed consent was obtained from all patients and the study was approved by the Regional Board of Research Ethics and the Norwegian Health authorities and conducted in accordance with the Helsinki declaration of 1971, as revised in 1983.

### Blood Sampling Procedures and Laboratory Measurements

Peripheral blood samples for determination of TnT, s-creatinine, s-glucose, s-lipids, hsCRP and BNP were drawn immediately following admission by direct venipuncture with a minimum of stasis of an antecubial vein. A repeated blood sample for the determination of TnT was drawn 7 hours following the primary blood sample. Clotted whole blood and ethylene diamine tetraacetic acid (EDTA) blood samples were centrifuged for 15 min with 2000 g and 20°C without delay. Serum for hsCRP and EDTA plasma for BNP were immediately frozen and stored at -80°C until the measurements were performed. For all other biochemical parameters, measurements were performed immediately following centrifugation.

BNP was analysed in EDTA plasma using the Microparticle Enzyme Immunoassay (MEIA) Abbott AxSYM^® ^(Abbott Laboratories, Abbott Park, Illinois, USA). The dynamic range was 0–4000 pg/mL and the within run coefficient of variation (CV) was 6.3% at 95 pg/mL and 4.7% at 1587 pg/mL.

HsCRP was measured with the use of an immunoturbidimetric assay (Tina-quant^® ^C-reactive protein (latex) high sensitive assay, Roche Diagnostics, Germany) performed on a Roche automated clinical chemistry analyzer (MODULAR P). The detection limit was 0.03 mg/L and the measuring range 0.1–20.0 mg/L with an extended measuring range with automatic re-run 0.1–300 mg/L. The between-assay CV was 3.45% at 1.19 mg/L and 2.70% at 0.43 mg/L.

TnT was quantified by a cardiac-specific second-generation troponin T ELISA assay from Roche diagnostics, using a high-affinity cardiac-specific TnT isoform antibody [15]. The lower detection limit of the assay used is 0.01 ng/mL. In this study a cut off level of 0.05 ng/mL was used with a CV of 10%.

### Statistical Analysis

The patients were divided into quartiles on the basis of their BNP and hsCRP levels. Approximately normally distributed variables were given as mean and standard deviation (SD), while variables with more skewed distributions were given as median and quartiles. The Chi-square test for association was applied between the BNP quartiles and categorical variables at baseline. The one-way ANOVA test was used to test for equality of means of scale variables (e.g. age), and the two-sample t test and Mann Whitney test were used for comparing the means and medians of two samples, respectively.

The hazard ratios (HR) are presented with 95% confidence interval (CI). A stepwise Cox multivariable proportional hazards regression model with total death, cardiac death and TnT positive events as the dependent variable, and BNP, hsCRP and other variables as potential independent predictors (listed below) was fitted. To examine the differences in prognosis between subjects in the upper versus the lower quartile(s) of BNP, we adjusted for sex, age, smoking, hypertension, index diagnosis, estimated glomerular filtration rate, diabetes mellitus, HF, history of previous CHD (i.e. history of either angina pectoris, MI, coronary artery bypass grafting or percutaneous coronary intervention), hypercholesterolemia, TnT > 0.05 ng/mL and hsCRP dichotomized at a cut point of 10 mg/L at admission and medication before enrollment (statins, clopidogrel, beta-blockers, ACE inhibitors and angiotensin receptor blockers). The Kaplan-Meier product limits were used for plotting the times to event.

In the discriminate analysis BNP, CRP, log_e_(BNP) and log_e_(CRP) were used as individual variables. The statistical analyses were performed using the statistical package SPSS version 15.0. All tests were two-sided with a significance level of 5%.

## Results

A total of 871 patients were enrolled in the RACS study. No BNP or hsCRP samples were available for 43 patients, thus 828 patients were included in the present analysis. No patient was lost to follow up. At index hospitalization, 367 patients (44.3%) had a peak TnT concentration exceeding 0.05 ng/mL. Eleven patients (2.8%) of the total population and 4 patients (3.2%) of the STEMI group presented with cardiogenic shock.

The baseline characteristics of the patients, stratified according to BNP quartiles at admission are listed in Table 1. The median BNP concentration in plasma was 97.0 (33.3–310.8) pg/mL [25 and 75% percentiles]. Patients with BNP in the higher quartiles were significantly older, had higher concentration of TnT exceeding 0.05 ng/mL, had diabetes mellitus, fewer were current smokers and they had a higher percentage of a previous history of heart failure, CVD and CHD. The time from symptom onset until blood sampling was 12 (6–38) hours [median, 25 and 75% percentiles].

**Table 1:** Baseline characteristics for patient strata according to quartiles of BNP

	**Quartiles of BNP, pg/mL**				
**Characteristics**	**< 33.0**	**33.0–96.0**	**> 96.0–310.0**	**> 310.0–4001.0**	**P value**
*Demographics*					
Age, y*	56.6 ± 13.4	67.8 ± 12.8	75.5 ± 10.7	78.4 ± 9.8	0.000
Female gender, % (n)	30.0 (62)	37.4 (77)	45.7 (95)	43.0 (89)	0.006
***Risk markers at baseline***					
TnT > 0.05 (ng/mL), % (n)	34.8 (72)	41.7 (86)	42.3 (88)	58.5 (121)	0.000
hsCRP* (mg/L)	7.3 ± 16.7	11.2 ± 31.8	21.5 ± 60.0	32.0 ± 66.7	0.000
***Risk factors***					
Hypercholesterolemia, % (n)	45.4 (94)	58.3 (120)	49.3 (102)	45.4 (94)	0.028
Total cholesterol (mmol/L)*	5.6 ± 1.2	5.2 ± 1.3	5.2 ± 1.3	4.9 ± 1.3	0.000
HDL cholesterol (mmol/L) *	1.3 ± 0.4	1.4 ± 0.5	1.4 ± 0.4	1.4 ± 0.5	0.305
Triglycerides (mmol/L) *	1.9 ± 1.6	1.6 ± 1.3	1.5 ± 0.9	1.4 ± 0.8	0.000
Current smoking, % (n)	41.5 (86)	25.2 (52)	19.2 (40)	19.3 (40)	0.000
Hypertension, % (n)	26.6 (55)	44.2 (91)	48.6 (101)	50.2 (104)	0.000
IDDM, % (n)	1.0 (2)	0.5 (1)	1.9 (4)	0.5 (1)	0.393
NIDDM, % (n)	5.8 (12)	14.1 (29)	14.4 (30)	15.5 (32)	0.010
eGFR* (ml/min/1.73 m^2^)	74.2 ± 17.6	66.3 ± 17.5	58.7 ± 20.3	50.9 ± 20.3	0.000
***History of heart failure***					
CHF, % (n)	6.8 (14)	14.6 (30)	27.9 (58)	57.5 (119)	0.000
NYHA (I/II/III/IV), n	2/8/4/0	5/18/6/1	9/30/18/1	13/51/53/2	0.000
***History of coronary heart disease***					
Angina pectoris, % (n)	30.9 (64)	42.7 (88)	48.1 (100)	52.7 (109)	0.000
MI, % (n)	15.0 (31)	30.1 (62)	38.5 (80)	48.3 (100)	0.000
CABG, % (n)	6.3(13)	12.1 (25)	9.6 (20)	12.6 (26)	0.128
PCI, % (n)	7.2 (15)	14.6 (30)	10.6 (22)	8.7 (18)	0.080
***Treatment prior to admission***					
Statins, % (n)	26.1 (54)	41.3 (85)	34.6 (72)	34.8 (72)	0.014
Clopidogrel, % (n)	1.0 (2)	2.9 (6)	1.0 (2)	2.9 (6)	0.249
β-blocade, % (n)	16.4 (34)	38.3 (79)	42.8 (89)	45.9 (95)	0.000
ACE inhibitors, % (n)	10.6 (22)	16.0 (33)	21.2 (44)	27.1 (56)	0.000
ARB, % (n)	6.3 (13)	19.4 (40)	17.3 (36)	20.8 (43)	0.000
Diuretics, % (n)	12.1 (25)	20.4 (42)	36.5 (76)	55.1 (114)	0.000
***Index diagnosis***					
STEMI, % (n)	18.4 (38)	18.4 (38)	11.1 (23)	11.6 (24)	0.042
NSTEMI, % (n)	15.9 (33)	23.8 (49)	31.3 (65)	46.9 (97)	0.000
UA, % (n)	2.4 (5)	4.9 (10)	13.9 (29)	15.5 (32)	0.000
No-ACS, % (n)	63.3 (131)	52.9 (109)	43.8 (91)	26.1 (54)	0.000

ACE; angiotensin converting enzyme, ARB; angiotensin receptor blockers, ACS; acute coronary syndrome, CABG; coronary artery bypass grafting, CHF; congestive heart failure, eGFR; estimated glomerular filtration rate, HDL; high-density lipoprotein, hsCRP; high-sensitive C-reactive protein, IDDM; insulin dependent diabetes mellitus, MI; myocardial infarction, NIDDM; non-insulin dependent diabetes mellitus, NYHA; New-York Heart Association functional class, STEMI; ST-segment elevation MI, NSTEMI; non ST-segment elevation MI, PCI; percutaneous coronary intervention, TnT; troponin T, UA; unstable angina pectoris. *Mean ± SD

BNP, CRP, log_e_(BNP) and log_e_(CRP) were used in the discriminate analysis as individual variables with the purpose to identify patients with true ACS versus non-cardiac chest pain and allocated 59.8%, 52.1%, 61.5% and 58.3%, respectively, of the observations correctly.

### All-Cause Mortality

**Figure 1:**
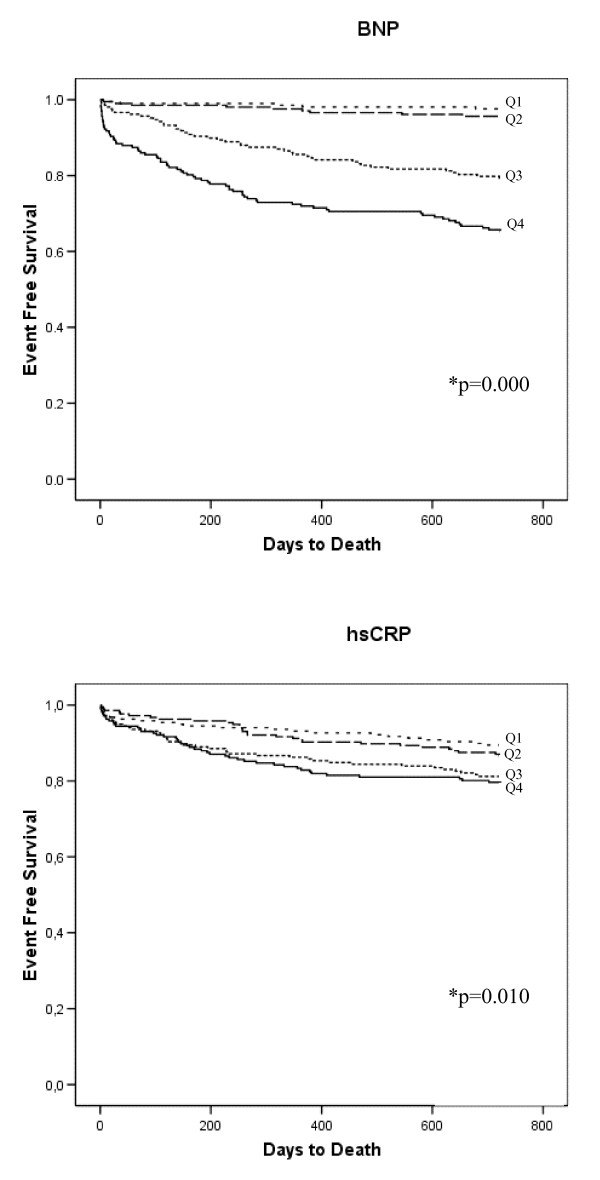
**Kaplan-Meier plots for the cumulative risk of the primary events (total mortality) for the BNP- and hsCRP quartiles, respectively**. *Log Rank (Mantel-Cox) test of equality of survival distribution for the different levels of the BNP and hsCRP quartiles (univariate analysis).

**Figure 2:**
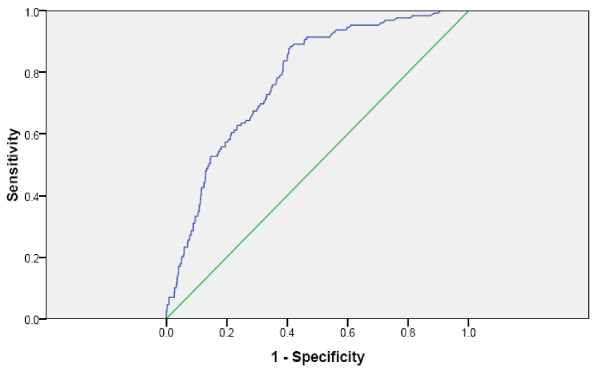
Receiver operated characteristics curve for BNP.

After a follow-up period of 24 months, 129 patients (15.6%) had died. Kaplan-Meier survival curves for the primary endpoint according to BNP and hsCRP quartiles at baseline are presented in Figure 1. The BNP levels were significantly higher among patients dying than in long-term survivors (401 (145–736) versus 75 (29–235) pq/mL [median, 25 and 75% percentiles], p = 0.000). Receiver operated characteristics (ROC) curve for BNP is shown in Figure 2; the area under the ROC for BNP was 0.778.

In a stepwise multivariable Cox regression model for death within 30 days and 2 years, the HR's for patients with BNP in the highest quartile (Q4) were 9.36 (95% CI, 1.10–79.83) and 5.13 (95% CI, 1.97–13.38), respectively, compared with Q1 (Table 2 &3). Within 2 years the HR was associated with all-cause mortality above and beyond age, CHF and the index diagnosis of STEMI (Table 3). In addition, in the subgroup composed of ACS patients, the adjusted HR within 2 years for the primary endpoint in patients with BNP in Q4 was 4.72 (95% CI, 1.76–12.68) compared with Q1 (Table 4).

**Table 2:** Cox regression model for the total population showing association between selected baseline clinical variables and hazards ratios for the prespecified endpoints during 30 days follow-up

***Variable***	Total Mortality*	Cardiac Death or Recurrent TnT Positive Cardiac Events*	Recurrent TnT Positive Cardiac Events*
	*HR (95% CI)*	*HR (95% CI)*	*HR (95% CI)*
BNP quartile 2	0.97 (0.09–11.07)	0.59 (0.20–1.74)	0.50 (0.15–1.74)
BNP quartile 3	3.49 (0.39–31.36)	1.63 (0.35–2.75)	0.52 (0.16–1.74)
BNP quartile 4	9.36 (1.10–79.83)	1.66 (0.60–4.55)	0.59 (0.18–1.97)
			
hsCRP quartile 2	0.25 (0.06–0.93)	1.01 (0.42–2.44)	9.94 (1.27–77.88)
hsCRP quartile 3	0.80 (0.31–2.02)	1.23 (0.53–2.88)	4.44 (0.51–38.57)
hsCRP quartile 4	0.71 (0.28–1.81)	1.55 (0.67–3.59)	14.79 (1.89–115.63)
			
Age	1.06 (1.01–1.11)	1.04 (1.01–1.07)	1.04 (1.00–1.08)
STEMI	0.19 (0.08–0.44)	0.41 (0.21–0.79)	
ARB	2.84 (1.27–6.40)		
TnT > 0.05 (ng/mL)	2.70 (1.06–6.84)	3.29 (1.62–6.65)	3.11 (1.41–6.88)
Hypertension		1.75 (1.01–3.03)	
Hypercholesterolemia			2.22 (1.05–4.68)

ARB; Angiotensin receptor blockers, BNP; B-type natriuretic peptide, CI; Confidence interval, CRP; C-reactive protein, HR; Hazard ratio, STEMI; Admission index diagnosis ST-segment elevation MI, TnT; Troponin T. *Multivariable Cox regression model for comparing quartiles versus the lower quartile

**Table 3:** Cox regression model for the total population showing association between selected baseline clinical variables and hazards ratios for the prespecified endpoints during 24 months follow-up

***Variable***	Total Mortality*	Cardiac Death or Recurrent TnT Positive Cardiac Events*	Recurrent TnT Positive Cardiac Events*
	*HR (95% CI)*	*HR (95% CI)*	*HR (95% CI)*
BNP quartile 2	1.02 (0.34–3.06)	0.87 (0.47–1.64)	0.97 (0.48–1.93)
BNP quartile 3	3.52 (1.35–9.19)	1.69 (0.94–3.04)	1.51 (0.78–2.95)
BNP quartile 4	5.13 (1.97–13.38)	2.30 (1.27–4.17)	1.60 (0.81–3.18)
			
hsCRP quartile 2	1.00 (0.56–1.78)	1.14 (0.73–1.79)	1.64 (0.98–2.73)
hsCRP quartile 3	1.23 (0.72–2.10)	1.04 (0.66–1.62)	1.04 (0.61–1.78)
hsCRP quartile 4	0.96 (0.56–1.65)	1.28 (0.83–1.97)	1.60 (0.96–2.66)
			
Age	1.07 (1.04–1.09)	1.04 (1.02–1.05)	1.02 (1.01–1.04)
NYHA 3			1.78 (1.10–2.89)
STEMI	0.41 (0.24–0.67)		
MI			1.68 (1.16–2.44)
Hypertension			1.67 (1.19–2.36)
NIDDM		1.54 (1.08–2.20)	1.66 (1.12–2.46)
CHF	1.57 (1.06–2.33)		
TnT>0.05 (ng/mL)		2.21 (1.64–3.00)	2.52 (1.78–3.56)
Angina pectoris			1.46 (1.02–2.09)
CHD		2.09 (1.39–3.13)	

BNP; B-type natriuretic peptide, CHD; Coronary heart disease, CI; Confidence interval, CHF; Congestive heart failure, CRP; C-reactive protein, HR; Hazard ratio, MI; Previous myocardial infarction, NIDDM; Non-insulin dependent diabetes mellitus, Non-ACS; NYHA; New-York Heart Association functional class, STEMI; Admission index diagnosis ST-segment elevation MI, TnT; Troponin T.

*Multivariable Cox regression model for comparing quartiles versus the lower quartile

The hsCRP levels were significantly higher among patients dying than in long-term survivors (6.6 (2.3–19.4) versus 3.6 (1.7–11.5) mg/L [median, 25 and 75% percentiles], p = 0.000). However, in the multivariable Cox regression model hsCRP rendered no prognostic information for all-cause mortality (Table 2 &3).

### Cardiac Death or Recurrent TnT Positive Cardiac Events

**Figure 3:**
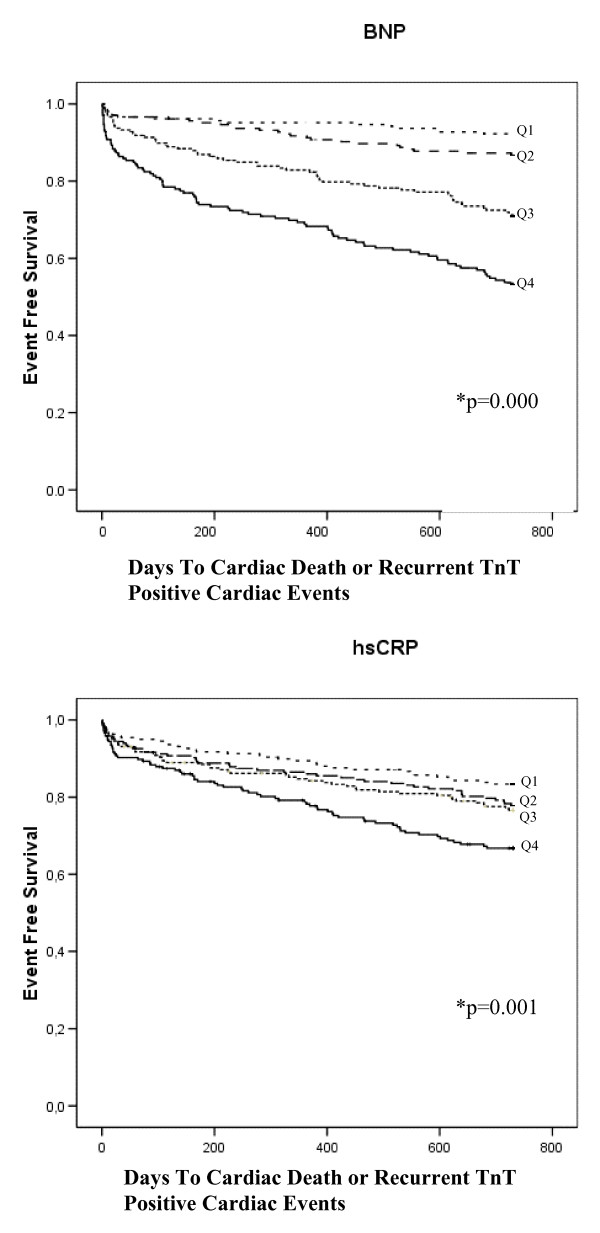
**Kaplan-Meier plots for the cumulative risk of cardiac death or recurrent TnT positive cardiac events for the BNP- and hsCRP quartiles, respectively**. *Log Rank (Mantel-Cox) test of equality of survival distribution for the different levels of the BNP and hsCRP quartiles (univariate analysis).

After a follow-up period of 24 months, 193 patients (23.3%) had experienced a cardiac death or a recurrent TnT positive cardiac event. Kaplan-Meier survival curves for the secondary endpoints are presented in Figure 3.

The adjusted HR for this combined endpoint within 2 years in patients with BNP in Q4 was 2.30 (95% CI, 1.27–4.17) compared with Q1 and was associated with the secondary endpoint above and beyond age, medical history of diabetes mellitus, CHD and TnT concentration at admission exceeding 0.05 ng/mL (Table 3). In addition, in the ACS subgroup, the adjusted HR for this secondary endpoint within 30 days and 2 years in patients with BNP in Q4 was 4.08 (95% CI, 1.67–9.98) and 2.33 (95% CI, 1.23–4.39), respectively, compared with Q1 (Table 4).

**Table 4:** Cox regression model for the acute coronary syndrome subgroup showing hazard ratios for the prespecified endpoints during 30 days and 24 months follow-up

***Variable***	Total Mortality*		Cardiac Death or Recurrent TnT Positive Cardiac Events*		Recurrent TnT Positive Cardiac Events*	
	***HR (95% CI)***		***HR (95% CI)***		***HR (95% CI)***	

	***30 days***	***24 months***	***30 days***	***24 months***	***30 days***	***24 months***
*BNP quartile 2*	1.01 (0.22–4.54)	2.33 (0.85–6.38)	0.98 (0.35–2.73)	1.40 (0.74–2.64)	0.42 (0.10–1.78)	1.29 (0.64–2.59)
*BNP quartile 3*	0.93 (0.20–4.48)	2.39 (0.88–6.54)	1.60 (0.59–4.33)	1.85 (0.99–3.48)	0.92 (0.28–3.04)	2.39 (1.24–4.61)
*BNP quartile 4*	3.83 (0.95–15.41)	4.72 (1.76–12.68)	4.08 (1.67–9.98)	2.33 (1.23–4.39)	1.08 (0.33–3.50)	1.88 (0.95–3.73)
*hsCRP quartile 2*	0.28 (0.06–1.33)	0.64 (0.33–1.21)	0.92 (0.35–2.39)	0.77 (0.46–1.27)	7.04 (0.86–57.41)	1.00 (0.57–1.76)
						
*hsCRP quartile 3*	1.98 (0.77–5.13)	1.27 (0.72–2.22)	1.78 (0.76–4.17)	0.95 (0.59–1.53)	4.02 (0.44–36.70)	0.89 (0.50–1.60)
*hsCRP quartile 4*	1.20 (0.45–3.18)	0.82 (0.46–1.44)	1.45 (0.61–3.42)	1.11 (0.70–1.76)	12.97 (1.63–103.10)	1.41 (0.83–2.39)

*Multivariable Cox regression model for comparing quartiles versus the lower quartile

The multivariable Cox regression model for a cardiac death or a recurrent TnT positive cardiac event within 30 days and 2 years in patients with hsCRP in Q4 compared with Q1 revealed no significant relation between this biomarkers and this secondary endpoint (Table 2 &3).

### Recurrent TnT Positive Cardiac Events

**Figure 4:**
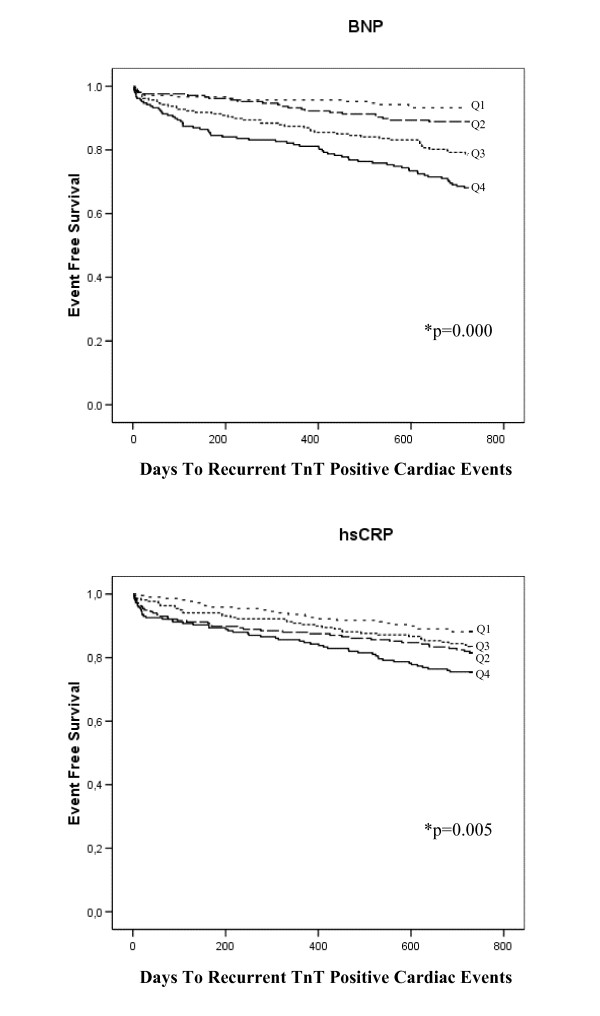
**Kaplan-Meier plots for the cumulative risk of recurrent TnT positive cardiac events for the BNP- and hsCRP quartiles, respectively**. *Log Rank (Mantel-Cox) test of equality of survival distribution for the different levels of the BNP and hsCRP quartiles (univariate analysis).

149 patients (18.0%) experienced a recurrent TnT positive cardiac event after a follow-up period of 2 years. Kaplan-Meier survival curves are presented in Figure 4.

The adjusted HR for this endpoint within 2 years in patients with BNP in Q4 was 1.60 (95% CI, 0.81–3.18) compared with Q1 and was not associated with a recurrent TnT positive cardiac event above and beyond age, HF, medical history of MI or angina pectoris, hypertension, diabetes mellitus and TnT values at admission (Table 3).

Within 30 days, the adjusted HR for patients with recurrent TnT cardiac positive events hsCRP in Q4 was 14.79 (95% CI, 1.89–115.63) compared with Q1 and was associated with this endpoint above and beyond age, hypercholesterolemia and TnT values at admission (Table 2). In addition, in the subgroup composed of ACS patients, the adjusted HR within 30 days in patients with hsCRP in Q4 was 12.97 (95% CI, 1.63–103.10) compared with Q1 (Table 4).

## Discussion

The main findings of our study in this group of unselected patients admitted to the ED with chest pain and potential ACS, indicate that BNP is an important and independent prognostic biomarker for both short- and long-term mortality. We could not confirm the prognostic ability of hsCRP as a predictor of mortality. However, hsCRP was found to be a predictor of short-term recurrent TnT positive cardiac events. Furthermore, the multivariable analysis showed that also some clinical variables at admission were all independent predictors of mortality. These latter results are in accordance with previous studies where several clinical background factors were associated with poor outcome [16-18]. However, after adjusting for these confounding factors, we found that the prognostic value of BNP and hsCRP were still statistically significant.

Given the heterogeneous nature of ACS, patients have a wide spectrum of risk for death and CV events. Our study was performed in a rather inhomogeneous and unselected patient population, but, importantly, it is similar to the one dealt with in the ED, reflecting the setting in the clinical every-day life.

In contrast to the majority of previous studies investigating the prognostic value of various biomarkers, our study had a prospective and observational design, and blood samples were collected directly on admission. Several other studies have measured natriuretic peptides levels within a few days from the onset of symptoms [4,6,20]. However, few studies have examined the predictive value of natriuretic peptides across the spectrum of ACS in blood samples obtained directly on admission before introducing any kind of therapy [19]. Therefore, in the present study we do not have to consider the potential confounding factors of late inclusions and recently introduced medical treatment; factors that need to be adjusted for when extracting prognostic information from randomized interventional studies.

Our data add to the discussion of the value of CRP as a predictive biomarker in patients with chest pain and ACS. Several previous studies have demonstrated an association between elevated CRP levels and increased probability of CHD events or death [18,21-23]. However, even if hsCRP was not a significant predictor of survival in our study it was shown to predict short-term recurrent TnT positive cardiac events.

CRP has been considered for adoption into risk assessment algorithms [24]. A study by Burke and colleagues supports the concept that hsCRP in serum reflects the numbers of vulnerable coronary atherosclerotic plaques with superficial foam cells and large necrotic cores in patients dying suddenly with CHD [25]. A natriuretic peptide system has also been detected in human coronary atherosclerotic plaques [26]. Interestingly, our study suggests that BNP is a better risk stratification tool for survival than CRP among patients with a potential ACS. There may be several potential explanations for the apparently more robust prognostic value of the natriuretic peptides, as they integrate multiple different pathophysiological signals within an individual, including ventricular dysfunction [27], elevated intracardiac filling pressures [1], valvular disease [28], ischemic burden [5] and diabetic nephropathy [29]. However, the exact underlying mechanisms of natriuretic peptides elevations in patients with ACS are not known at present.

Recently conducted studies support our results and show that the independent predictivity of CRP was attenuated when it was tested in the multivariable model in the general population [30] and together with natriuretic peptides in patients with known CAD [31-33]. These divergent findings with respect to the positive predictivity of CRP might be explained by factors such as differences in the patients' characteristics, duration of follow-up, definitions of endpoints and by the fact that CRP is more closely related to CV risk factors than to CV damage [30].

Further studies are needed to identify therapies that may reduce the risk associated with increased BNP or hsCRP. We would assume that an early risk stratification of our patient population presenting to the ED could be important for several reasons. The finding of an early rise in the natriuretic peptides obtained immediately after presenting to the ED with an ACS might be favourably influenced by immediate or early interventions [34]. However, there have been conflicting results regarding this topic [5]. The observation in our study that BNP did not predict recurrent TnT positive cardiac events is in accordance with prior studies [4,33]. Patients at low risk, as defined by low natriuretic peptides values in combination with normal troponins may benefit more from a conservative management [34]. Thus, additional evidence regarding optimal decision limits, use in combination with troponins and use in targeting therapy is needed before acceptance into clinical use for ACS.

### Limitations

The potential limitations of these data merit consideration. This study was conducted at a single-center, but patients were unselected and characterized by generally accepted diagnostic criteria, including their TnT values. The mortality rates in the present investigation were high; 129 patients (15.6%) at 24 months, most likely due to an unselected, consecutive recruitment, resulting in a high percentage of elderly patients and patients with comorbidity. The increased long-term mortality was associated with elevated BNP concentrations, pointing to the need for further investigations into the underlying pathomechanisms of BNP release. However, we believe that these features are common among patients with chest pain and potential ACS and that the results are generalizable. The circulating concentrations of BNP and CRP prior to admission remain unknown and our analyses are based on a single baseline determination. Although we did not adjust for LVEF, we did adjust for known CHF and CVD, including previous MI, and other clinical risk factors.

## Conclusion

BNP may act as a clinically useful biomarker when obtained at admission in an unselected patient population following hospitalization with chest pain and potential ACS, and may provide complementary prognostic information to established risk determinants at long-term follow-up. Our data do not support the hypothesis that the additional assessment of hsCRP will lead to better risk stratification for survival than BNP alone.

## Competing interests

David Pritchard is employed at Axis-Shield, United Kingdom.

## Authors' contributions

TBA: Contributed to the follow-up of the patients, interpretation of the results and wrote the first draft of the manuscript. VP: Investigated the patients and commented on the manuscript. HS: Performed the statistical analysis and contributed to the interpretation of the results. DP: Carried out the immunoassays. HG: Participated in the design, investigated the patients and commented on the manuscript. DWTN: Conceived the idea of the study, supervised the study and commented on the manuscript with important intellectual content.

All authors have read and approved the final manuscript.

## Pre-publication history

The pre-publication history for this paper can be accessed here:



## References

[B1] Maeda K, Tsutamoto T, Wada A, Hisanaga T, Kinoshita M (1998). Plasma brain natriuretic peptide as a biochemical marker of high left ventricular end-diastolic pressure in patients with symptomatic left ventricular dysfunction. Am Heart J.

[B2] Nakagawa O, Ogawa Y, Itoh H, Suga S, Komatsu Y, Kishimoto I, Nishino K, Yoshimasa T, Nakao K (1995). Rapid transcriptional activation and early mRNA turnover of brain natriuretic peptide in cardiocyte hypertrophy. Evidence for brain natriuretic peptide as an "emergency" cardiac hormone against ventricular overload. J Clin Invest.

[B3] Richards AM, Nicholls MG, Espiner EA, Lainchbury JG, Troughton RW, Elliott J, Frampton C, Turner J, Crozier IG, Yandle TG (2003). B-type natriuretic peptides and ejection fraction for prognosis after myocardial infarction. Circulation.

[B4] de Lemos JA, Morrow DA, Bentley JH, Omland T, Sabatine MS, McCabe CH, Hall C, Cannon CP, Braunwald E (2001). The prognostic value of B-type natriuretic peptide in patients with acute coronary syndromes. N Engl J Med.

[B5] Morrow DA, de Lemos JA, Sabatine MS, Murphy SA, Demopoulos LA, Dibattiste PM, McCabe CH, Gibson CM, Cannon CP, Braunwald E (2003). Evaluation of B-type natriuretic peptide for risk assessment in unstable angina/non-ST-elevation myocardial infarction: B-type natriuretic peptide and prognosis in TACTICS-TIMI 18. J Am Coll Cardiol.

[B6] Omland T, Persson A, Ng L, O'Brien R, Karlsson T, Herlitz J, Hartford M, Caidahl K (2002). N-terminal pro-B-type natriuretic peptide and long-term mortality in acute coronary syndromes. Circulation.

[B7] Ross R (1999). Atherosclerosis–an inflammatory disease. N Engl J Med.

[B8] Liuzzo G, Biasucci LM, Gallimore JR, Grillo RL, Rebuzzi AG, Pepys MB, Maseri A (1994). The prognostic value of C-reactive protein and serum amyloid a protein in severe unstable angina. N Engl J Med.

[B9] Berk BC, Weintraub WS, Alexander RW (1990). Elevation of C-reactive protein in "active" coronary artery disease. Am J Cardiol.

[B10] de Lemos JA (2006). The latest and greatest new biomarkers: which ones should we measure for risk prediction in our practice?. Arch Intern Med.

[B11] Rothenbacher D, Koenig W, Brenner H (2006). Comparison of N-terminal pro-B-natriuretic peptide, C-reactive protein, and creatinine clearance for prognosis in patients with known coronary heart disease. Arch Intern Med.

[B12] Kim H, Yang DH, Park Y, Han J, Lee H, Kang H, Park HS, Cho Y, Chae SC, Jun JE, Park WH (2006). Incremental prognostic value of C-reactive protein and N-terminal proB-type natriuretic peptide in acute coronary syndrome. Circ J.

[B13] Sabatine MS, Morrow DA, de Lemos JA, Gibson CM, Murphy SA, Rifai N, McCabe C, Antman EM, Cannon CP, Braunwald E (2002). Multimarker approach to risk stratification in non-ST elevation acute coronary syndromes: simultaneous assessment of troponin I, C-reactive protein, and B-type natriuretic peptide. Circulation.

[B14] Ndrepepa G, Kastrati A, Braun S, Mehilli J, Niemoller K, von BN, von BO, Vogt W, Schomig A (2006). N-terminal probrain natriuretic peptide and C-reactive protein in stable coronary heart disease. Am J Med.

[B15] Muller-Bardorff M, Hallermayer K, Schroder A, Ebert C, Borgya A, Gerhardt W, Remppis A, Zehelein J, Katus HA (1997). Improved troponin T ELISA specific for cardiac troponin T isoform: assay development and analytical and clinical validation. Clin Chem.

[B16] Antman EM, Cohen M, Bernink PJ, McCabe CH, Horacek T, Papuchis G, Mautner B, Corbalan R, Radley D, Braunwald E (2000). The TIMI risk score for unstable angina/non-ST elevation MI: A method for prognostication and therapeutic decision making. JAMA.

[B17] Boersma E, Pieper KS, Steyerberg EW, Wilcox RG, Chang WC, Lee KL, Akkerhuis KM, Harrington RA, Deckers JW, Armstrong PW, Lincoff AM, Califf RM, Topol EJ, Simoons ML (2000). Predictors of outcome in patients with acute coronary syndromes without persistent ST-segment elevation. Results from an international trial of 9461 patients. The PURSUIT Investigators. Circulation.

[B18] Lindahl B, Toss H, Siegbahn A, Venge P, Wallentin L (2000). Markers of myocardial damage and inflammation in relation to long-term mortality in unstable coronary artery disease. FRISC Study Group. Fragmin during Instability in Coronary Artery Disease. N Engl J Med.

[B19] Jernberg T, Stridsberg M, Venge P, Lindahl B (2002). N-terminal pro brain natriuretic peptide on admission for early risk stratification of patients with chest pain and no ST-segment elevation. J Am Coll Cardiol.

[B20] Omland T, de Lemos JA, Morrow DA, Antman EM, Cannon CP, Hall C, Braunwald E (2002). Prognostic value of N-terminal pro-atrial and pro-brain natriuretic peptide in patients with acute coronary syndromes. Am J Cardiol.

[B21] Ballantyne CM, Nambi V (2005). Markers of inflammation and their clinical significance. Atheroscler Suppl.

[B22] Kavsak PA, MacRae AR, Newman AM, Lustig V, Palomaki GE, Ko DT, Tu JV, Jaffe AS (2007). Elevated C-reactive protein in acute coronary syndrome presentation is an independent predictor of long-term mortality and heart failure. Clin Biochem.

[B23] Toss H, Lindahl B, Siegbahn A, Wallentin L (1997). Prognostic influence of increased fibrinogen and C-reactive protein levels in unstable coronary artery disease. FRISC Study Group. Fragmin during Instability in Coronary Artery Disease. Circulation.

[B24] Ridker PM (2003). Clinical application of C-reactive protein for cardiovascular disease detection and prevention. Circulation.

[B25] Burke AP, Tracy RP, Kolodgie F, Malcom GT, Zieske A, Kutys R, Pestaner J, Smialek J, Virmani R (2002). Elevated C-reactive protein values and atherosclerosis in sudden coronary death: association with different pathologies. Circulation.

[B26] Casco VH, Veinot JP, Kuroski de Bold ML, Masters RG, Stevenson MM, de Bold AJ (2002). Natriuretic peptide system gene expression in human coronary arteries. J Histochem Cytochem.

[B27] Maisel AS, Krishnaswamy P, Nowak RM, McCord J, Hollander JE, Duc P, Omland T, Storrow AB, Abraham WT, Wu AH, Clopton P, Steg PG, Westheim A, Knudsen CW, Perez A, Kazanegra R, Herrmann HC, McCullough PA (2002). Rapid measurement of B-type natriuretic peptide in the emergency diagnosis of heart failure. N Engl J Med.

[B28] Gerber IL, Stewart RA, Legget ME, West TM, French RL, Sutton TM, Yandle TG, French JK, Richards AM, White HD (2003). Increased plasma natriuretic peptide levels reflect symptom onset in aortic stenosis. Circulation.

[B29] Beer S, Golay S, Bardy D, Feihl F, Gaillard RC, Bachmann C, Waeber B, Ruiz J (2005). Increased plasma levels of N-terminal brain natriuretic peptide (NT-proBNP) in type 2 diabetic patients with vascular complications. Diabetes Metab.

[B30] Olsen MH, Christensen MK, Hansen TW, Gustafsson F, Rasmussen S, Wachtell K, Borch-Johnsen K, Ibsen H, Jorgensen T, Hildebrandt P (2006). High-sensitivity C-reactive protein is only weakly related to cardiovascular damage after adjustment for traditional cardiovascular risk factors. J Hypertens.

[B31] de Winter RJ, Stroobants A, Koch KT, Bax M, Schotborgh CE, Mulder KJ, Sanders GT, van Straalen JP, Fischer J, Tijssen JG, Piek JJ (2004). Plasma N-terminal pro-B-type natriuretic peptide for prediction of death or nonfatal myocardial infarction following percutaneous coronary intervention. Am J Cardiol.

[B32] Schnabel R, Rupprecht HJ, Lackner KJ, Lubos E, Bickel C, Meyer J, Munzel T, Cambien F, Tiret L, Blankenberg S (2005). Analysis of N-terminal-pro-brain natriuretic peptide and C-reactive protein for risk stratification in stable and unstable coronary artery disease: results from the AtheroGene study. Eur Heart J.

[B33] James SK, Lindahl B, Siegbahn A, Stridsberg M, Venge P, Armstrong P, Barnathan ES, Califf R, Topol EJ, Simoons ML, Wallentin L (2003). N-terminal pro-brain natriuretic peptide and other risk markers for the separate prediction of mortality and subsequent myocardial infarction in patients with unstable coronary artery disease: a Global Utilization of Strategies To Open occluded arteries (GUSTO)-IV substudy. Circulation.

[B34] James SK, Lindback J, Tilly J, Siegbahn A, Venge P, Armstrong P, Califf R, Simoons ML, Wallentin L, Lindahl B (2006). Troponin-T and N-terminal pro-B-type natriuretic peptide predict mortality benefit from coronary revascularization in acute coronary syndromes: a GUSTO-IV substudy. J Am Coll Cardiol.

